# Homework adherence in exposure-based CBT for youth with obsessive-compulsive disorder: Clinical outcomes and predictors across treatment

**DOI:** 10.1016/j.brat.2026.105050

**Published:** 2026-04-20

**Authors:** Abigail E. Pine, Qimin Liu, Eric A. Storch, Daniel A. Geller, Brent J. Small, Sabine Wilhelm, Joseph F. McGuire

**Affiliations:** a Division of Child & Adolescent Psychiatry, Department of Psychiatry and Behavioral Sciences, Johns Hopkins University School of Medicine, Baltimore, MD, USA; b Developmental Behavioral Health, Kennedy Krieger Institute, Baltimore, MD, USA; c Department of Psychological and Brain Sciences, Boston University, Boston, MA, USA; d Department of Psychiatry and Behavioral Sciences, Baylor College of Medicine, Houston, TX, USA; e Massachusetts General Hospital, Boston, MA, USA; f Harvard University Medical School, Boston, MA, USA; g University of North Carolina, Chapel Hill, NC, USA

**Keywords:** Pediatric OCD, Homework adherence, Exposure-based CBT

## Abstract

Exposure-based cognitive behavioral therapy (CBT) is the frontline treatment for pediatric obsessive-compulsive disorder (OCD), but not all youth fully respond to this treatment. While multiple factors may influence CBT response, homework adherence in CBT is a modifiable target that can improve treatment outcomes. This report examines the relationship between homework adherence and clinical outcomes in a large sample of youth with OCD who received exposure-based CBT. Here, 137 youth with OCD between 7 and 17 years old (*M* = 12.42, *SD* = 2.88) participated in a randomized controlled trial of exposure-based CBT. Homework adherence was monitored weekly, and OCD severity was assessed by independent evaluators masked to treatment condition using gold-standard rating scales. Mixed-effects linear and logistic regression models examined the relationship between homework adherence, reductions in OCD severity, treatment response, and clinical remission at post-treatment. Follow-up investigations explored differences in patterns between early- and late-homework adherence. Finally, baseline clinical predictors of homework adherence were explored. There was a significant predictive relationship between greater homework adherence and reduced OCD severity, greater incidence of treatment response, and greater incidence of clinical remission at post-treatment. Greater homework adherence later in treatment—as opposed to earlier in treatment—was most impactful in predicting positive clinical outcomes in exposure-based CBT. Presence of co-occurring ADHD was a significant predictor of decreased homework adherence. Taken together, findings provide insight into a modifiable therapeutic target that can improve treatment outcomes in exposure-based CBT.

Obsessive-compulsive disorder (OCD) affects up to 3% of the population, and predominantly onsets in childhood (Ruscio et al., 2010; Zohar, 1999). Its symptoms cause significant functional impairment across life domains (e.g., social, academic, and family functioning; Piacentini et al., 2007) and persist into adulthood if left untreated (Micali et al., 2010; Wewetzer et al., 2001). Fortunately, two evidenced-based interventions exist. Exposure-based cognitive behavioral therapy (CBT)—either alone or in combination with medication—is recommended as the frontline intervention for pediatric OCD (Abramowitz et al., 2005; Franklin et al., 2011; Freeman et al., 2014). Unfortunately, up to 50% of youth do not fully respond to CBT for several reasons (e.g., partial and/or non-responders; Kemp et al., 2021; McGuire et al., 2015). Thus, there is a clear need to better identify and optimize modifiable targets to improve the efficacy of this frontline intervention.

While some debate remains about the optimal targets for exposure (e.g., habituation versus inhibitory learning; Benito et al., 2021; Guzick et al., 2020; Kircanski et al., 2014; Kircanski & Peris, 2015), evidence shows that greater completion of exposures in CBT sessions results in greater symptom severity reductions (Peris et al., 2015, 2017). In CBT for OCD, youth typically receive one weekly 60-minute session that is supplemented by several hours of assigned exposure “homework” on multiple days between CBT sessions. Thus, most exposures in CBT are completed between sessions as “homework” rather than within sessions themselves. However to date, only a handful of studies have examined the relationship between homework adherence in CBT and treatment outcomes in adults (Conklin et al., 2021; Wheaton & Chen, 2021) and youth with OCD (Olatunji et al., 2015; Park et al., 2014; Selles et al., 2018; Tuerk et al., 2024; Walther et al., 2022). These reports found significant associations between greater homework adherence in CBT and reductions in OCD severity. While these findings highlight the importance of homework adherence in CBT, several limitations exist and warrant consideration before making broad extrapolation of findings.

First and foremost, while adult studies have had relatively large sample sizes (28 to 59 participants), studies investigating homework adherence among youth receiving individual treatment have had much more modest sample sizes (27 to 30 participants). Drawing clinical conclusions based on limited sample sizes can be problematic, as smaller samples result in both decreased power and probability that a significant finding is indeed true (Ioannidis, 2005). Second, there remain questions about when homework adherence in CBT may be most impactful. For instance, prior work on homework adherence in both youth and adults has been inconsistent as to whether adherence during early or late stages of exposure-based CBT is most critical in predicting positive outcomes (De Araujo et al., 1996; Walther et al., 2022; Wheaton & Chen, 2021). While it is possible that setting up patients early for a trajectory of homework success is beneficial (De Araujo et al., 1996; Wheaton & Chen, 2021), it is also possible that adherence is most important during specific therapeutic content such as exposures (Walther et al., 2022). Clarifying the impact of homework adherence throughout different phases of treatment can inform when therapists choose to emphasize the importance of its completion. Third, existing studies have solely examined the impact of homework adherence on OCD severity reductions. Significant symptom reduction does not necessarily translate to clinically meaningful improvement (Nordahl-Hansen et al., 2018), and as such, additional examination of the role of homework adherence in predicting treatment response and OCD remission is warranted.

Finally, prior investigations have largely focused on factors that predict greater homework adherence and positive treatment outcomes. However, it is equally important to identify and understand which patients may require greater support to improve homework adherence in CBT. In adults, the clinical predictors most consistently associated with homework adherence include greater insight into OCD symptoms, reduced OCD severity, greater treatment readiness, and less avoidance (De Araujo et al., 1996; Dowling et al., 2016; Wheaton & Chen, 2021). Meanwhile, only one report in youth with OCD has examined this topic and found that baseline externalizing symptoms predicted homework adherence—whereas baseline OCD severity and depression did not (Park et al., 2014). Given the broad domain of “externalizing symptoms” and inconsistencies across reports, further investigation is essential to determine the characteristics of participants who may have low homework adherence. This information can help therapists identify patients who may be at greater risk of low homework adherence in treatment—which in turn can guide treatment planning and/or personalized interventions to optimize homework adherence in CBT.

To address these identified gaps in the literature, this paper examines the relationship between homework adherence and clinical treatment outcomes in a large sample of youth with OCD (*n* = 137) who participated in a randomized controlled trial of exposure-based CBT. First, we examined the association between homework adherence and treatment outcomes, focusing on reductions in OCD severity, treatment response, and clinical remission. Based on the extant literature, we hypothesized that greater homework adherence will significantly predict fewer symptoms and greater likelihood of positive treatment response and remission following treatment. Second, we investigated whether early versus late adherence is most salient in predicting positive treatment outcomes. Given prior conflicting findings (Walther et al., 2022; Wheaton & Chen, 2021), we did not have any a priori hypotheses on timing of homework adherence in predicting outcomes. Lastly, given the clear need to identify characteristics of patients who may have lower homework adherence, we explored whether depression symptoms, OCD symptoms severity, family accommodation, ADHD, and lower insight predict overall homework adherence. We hypothesized that greater depression symptoms, greater OCD symptom severity, higher family accommodation, a diagnosis of ADHD, and lower insight will predict less overall homework adherence.

## 1. Method

### 1.1. Participants

Participants included 137 youth between the ages of 7 and 17 years old (*M* = 12.42, *SD* = 2.88) who completed all clinician administered OCD assessments and CBT visits as part of a multi-site randomized controlled trial examining the benefit of d-cycloserine (DCS) or placebo in exposure-based CBT (Storch et al., 2016). Inclusion criteria were a current and primary DSM-IV-TR OCD diagnosis determined via the Schedule for Affective Disorders and Schizophrenia for School-Age Children—Present and Lifetime version (K-SADS-PL; Kaufman et al., 1997), a Total Score of at least 16 on the Children’s Yale-Brown Obsessive Compulsive Scale (CY-BOCS; Scahill et al., 1997), as well as a full-scale IQ of at least 85 (Wechsler, 1999). Youth were excluded if: (1) they started an antidepressant or antipsychotic medication within 12 or 6 weeks, respectively, or had a recent increase in dosage; (2) a contraindication to the DCS medication was present (e.g., epilepsy diagnosis, renal insufficiency, DCS allergy); (3) they were not able to swallow study medication; (4) they had active suicidality or a suicide attempt within the past year; (5) they had comorbid diagnoses of psychosis, bipolar disorder, autistic disorder, anorexia nervosa, or non-OCD primary hoarding symptoms. Additional details on study participants and criteria is discussed elsewhere (Storch et al., 2016).

### 1.2. Measures

#### K-SADS-PL (Kaufman et al., 1997).

The K-SADS-PL is a clinician administered semi-structured diagnostic interview given at screening to assess DSM-IV childhood disorders, including those utilized to determine study eligibility. It has demonstrated good reliability and validity in prior samples of same age youth (Kaufman et al., 1997).

#### Homework Adherence.

Homework adherence quality and quantity was assessed by clinician rating at Sessions 2–10. Clinicians asked questions during the session following the homework assignment to determine a homework adherence score on a 7-point Likert scale that ranged from 0 (“*did not complete any assigned homework*”) to 6 (“*completed all homework assignments and made efforts above and beyond assignments*”). This rating system has been used successfully to assess homework adherence in other trials (Park et al., 2014; Storch, Murphy, et al., 2010). Importantly, given prior work suggesting the importance of early versus late adherence (Walther et al., 2022), a subset of analyses examined early (average across sessions 2–5) and late (average across sessions 6–10) adherence separately.

#### CY-BOCS (Scahill et al., 1997).

The CY-BOCS is a semi-structured clinician-administered interview that examines the presence and severity of OCD symptoms over the past week. The CY-BOCS total score consisting of 10-items has shown good reliability, validity, and treatment sensitivity (Scahill et al., 1997; Storch et al., 2004; Storch, Lewin, et al., 2010). The CY-BOCS item 11 is a measure of clinician rated participant insight, ranging from 0 (“*excellent insight”*) to 4 (“*lacks insight*”). The CY-BOCS was administered at baseline, sessions 2, 4, 5, 7, 9, 10, and post-treatment. A score of ≤12 was utilized to categorize OCD remission at post-treatment (Farris et al., 2013; Mataix-Cols et al., 2016; Storch et al., 2016). Internal consistency across assessments for the CY-BOCS in the current sample was α = .84–0.92.

#### Clinical Global Impression – Improvement (CGI-I; Guy, 1976).

The CGI-I is a 7-point rating indicating level of treatment response. It is a well-validated clinician rated score ranging from 1 (“*very much improved*”) to 7 (“*very much worse*”). A CGI-I score was given at the post-treatment assessment to characterize treatment response since treatment onset. A positive treatment response was characterized by scores of *very much* (1) and *much improved* (2) (Skarphedinsson et al., 2017; Storch, Lewin, et al., 2010).

#### Children’s Depression Rating Scale, Revised (CDRS-R; Poznanski & Mokros, 1996).

The CDRS-R is a semi-structured clinical interview assessing depression severity in youth. This measure was administered at baseline and has demonstrated good reliability and validity for use among children and adolescents (Jain et al., 2007; Mayes et al., 2010). Internal consistency in the current sample for the CDRS-R was α = .85.

#### Family Accommodation Scale-Interview Rated (FAS; Calvocoressi et al., 1999).

The FAS examines parental accommodation of obsessive-compulsive symptoms. It assesses 12 different accommodation behaviors and has evidenced excellent reliability and validity (Calvocoressi et al., 1999). The FAS was administered at baseline. Internal consistency for the FAS in the current sample was α = .76.

### 1.3. Procedures

All study procedures were approved by the institutional review boards at the two recruitment sites. Written parental consent and youth assent were obtained for all participants. Both the K-SADS-PL and CY-BOCS were administered to determine study eligibility at an initial screening appointment. Exposure-based CBT occurred across 10 sessions over 8 weeks. Sessions 1–3 covered psychoeducation, hierarchy development, and cognitive therapy, and exposure therapy began in session 4. Youth were randomized prior to the fourth CBT session to either a DCS or placebo condition. The current study includes participants from both groups as all youth, regardless of condition assignment, received exposure-based CBT. A post-treatment assessment was completed following the final CBT session. Additional study procedures and primary trial outcomes are reported elsewhere (Storch et al., 2016).

### 1.4. Analytic plan

All analyses were conducted using the *lme4* package (Bates et al., 2015) in R (R Core Team, 2024). Four participants had missing homework adherence data and were therefore excluded from analyses. An independent samples *t*-test was performed to examine differences in homework adherence based upon condition assignment.

To account for the nested structure of repeated measures data, three mixed-effects linear models were fitted to predict continuous outcomes. The first model examined the relationship between homework adherence and CY-BOCS outcomes. This model included the following predictors: (1) average homework adherence across all sessions, (2) person mean-centered homework adherence at each session, (3) session number, (4) the interaction between session and average homework adherence, and (5) the interaction between session and person-mean centered homework adherence. A second mixed-effects linear model was fitted to predict CY-BOCS scores while examining early and late homework adherence separately. Predictors for this model included: (1) average homework adherence across sessions 2–5 (early adherence), (2) average homework adherence across sessions 6–10 (late adherence), (3) session number, (4) the interaction between session and early adherence, and (5) the interaction between session and late adherence. Because early and late homework adherence were included simultaneously in the model, multicollinearity was evaluated using variance inflation factors (VIFs). In all models, random intercepts and slopes for session were included at the participant level to account for individual variability in symptom and treatment response trajectories. Sensitivity analyses were conducted by re-estimating models with additional covariates including parent-reported youth academic performance and parental educational attainment.

Two logistic regressions were conducted to examine associations between homework adherence and dichotomous outcomes: (1) a positive treatment response and (2) OCD remission, defined as CY-BOCS ≤12 following intervention. A final mixed-effects linear model was used to examine predictors of homework adherence across treatment. Predictors included treatment session number, baseline CDRS-R, baseline CY-BOCS, age, sex, baseline FAS, ADHD diagnosis, and baseline OCD insight. A random intercept for participant was included to account for the nested structure of the data. A random slope for treatment session number was not included in the final model given nonconvergence. All mixed-effects models were estimated using full information maximum likelihood. To account for multiple analyses, the Benjamini-Hochberg (BH procedure (Benjamini & Hochberg, 1995) was utilized to control the false discovery rate (FDR) at α = .05.

## 2. Results

Descriptive statistics and zero-order correlations among key study variables are reported in Table 1. There were no significant differences in average homework adherence based upon treatment condition assignment (DCS: *M* = 4.11, *SD* = 0.83; placebo: *M* = 4.09, *SD* = 1.08), *t* (131) = 0.10, Cohen’s *d* = 0.02, 95% CI [−0.31, 0.35], *p* > .05.

### 2.1. Homework adherence and OCD symptoms

A mixed-effects linear model was fitted to predict CY-BOCS total scores, and model results are presented in Table 2. All main effects in this model, including person mean-centered homework adherence (*β* = 0.05, 95% CI [−0.03, 0.14], *p* = .23), mean homework adherence (*β* = −0.03, 95% CI [−0.19, 0.12], *p* = .68), and CBT session (*β* = −0.20, 95% CI [−0.44, 0.05], *p* = .12) were not significant. However, both interactions in the model were significant. Specifically, the interaction between person mean-centered homework adherence and session was significant (*β* = −0.05, 95% CI [−0.09, −0.01], *p* = .02), which suggests that within-person increases in homework adherence were associated with a decreasing trend in OCD severity across treatment. Additionally, the interaction between mean homework adherence and session was significant (*β* = −0.07, 95% CI [−0.13, −0.01], *p* = .02), which indicates that participants with higher average homework adherence showed decreasing trends in OCD severity across treatment.

**Fig. 1.**
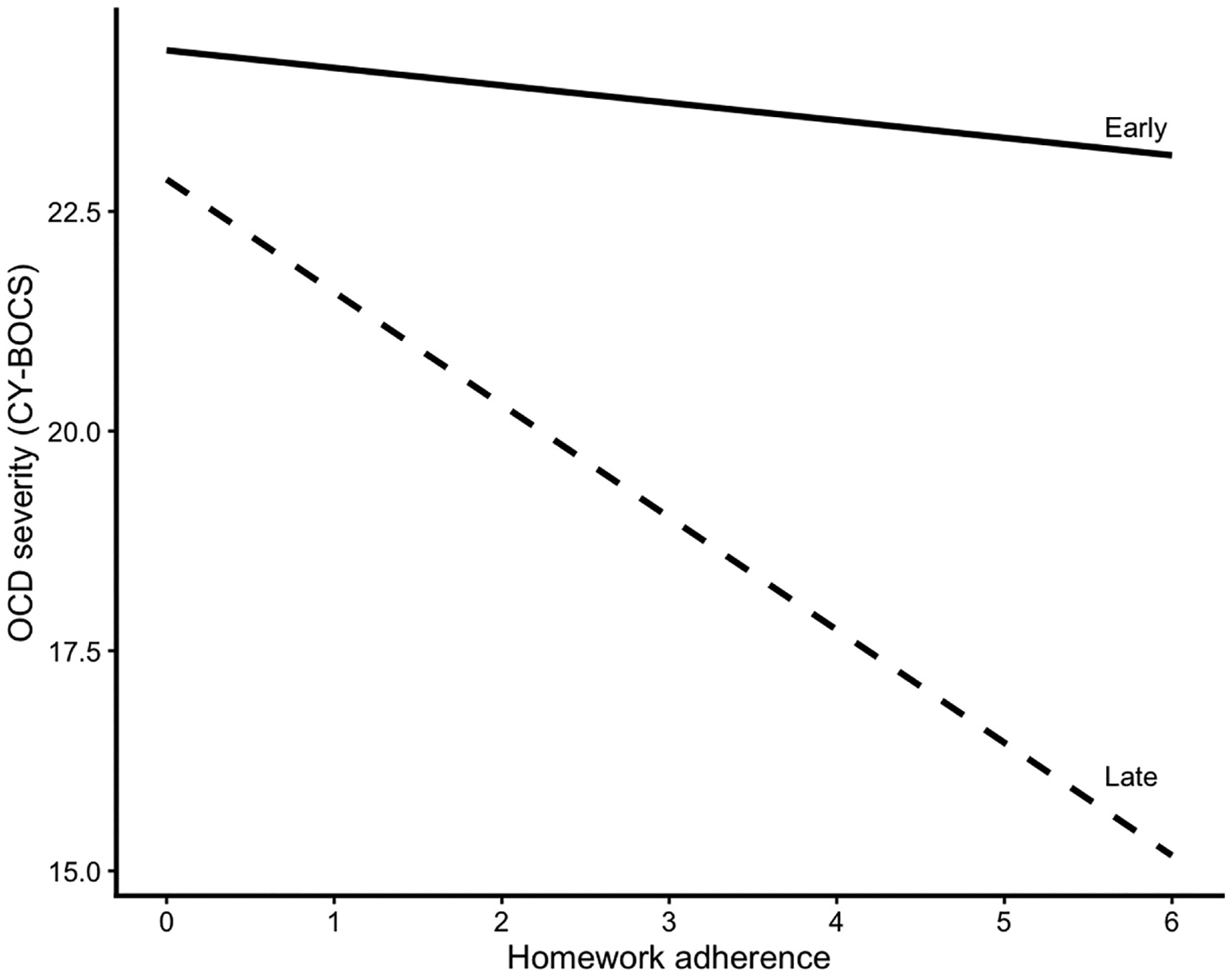
Association between session-level homework adherence and OCD symptom severity across treatment. Lines depict estimated linear relationships between homework adherence and CY-BOCS scores during early (sessions 2–5) and late (sessions 6–10) treatment phases.

A second mixed-effects linear model examined the effects of early and late homework adherence on CY-BOCS total scores, and results from this model are presented in Table 3. VIF values for early and late adherence were both 2.82, indicating acceptable levels of collinearity. Main effects for early (β = −0.10, 95% CI [−0.28, 0.08], p = .29) and late (β = 0.02, 95% CI [−0.16, 0.21], p = .81) adherence were not significant. Session was significantly associated with CY-BOCS scores (β = −0.27, 95% CI [−0.51, −0.03], p = .03), which indicates improvement in OCD severity across treatment. The interaction between session and early adherence was not significant (β = 0.06, 95% CI [−0.01, 0.13], p = .07), which suggests that early adherence was not associated with changes in OCD severity across treatment. By contrast, the interaction between session and late adherence was significant (β = −0.13, 95% CI [−0.20, 0.06], p < .001) that indicates that higher adherence later in treatment was associated with greater improvement in OCD severity across sessions. Model results were unchanged after adjusting for youth academic performance and parental educational attainment. A visual representation of the estimated association between session-level homework adherence and OCD symptom severity across early and late phases of treatment is presented in Fig. 1.

### 2.2. Homework adherence, treatment response, and remission

Logistic regression analyses revealed that greater average homework adherence was significantly associated with an increased odds of a positive treatment response, B = 0.87, *p* = .002, OR = 2.38, 95% CI [1.36, 4.16]. Greater average homework adherence was also significantly associated with an increased odds of clinical remission at post-treatment, B = 0.53, *p* = .008, OR = 1.71, 95% CI [1.15, 2.53].

### 2.3. Predictors of homework adherence

Findings from the linear mixed-effects model examining predictors of homework adherence indicated that ADHD diagnosis was associated with decreased homework adherence (*β* = −0.14, 95% CI: [−0.26, −0.02], *p* = .02), with participants diagnosed with ADHD demonstrating significantly lower homework adherence. Additionally, sex was a significant predictor (*β* = −0.12, 95% CI [−0.23, −0.01], *p* = .04), indicating that males exhibited lower homework adherence when compared to females. However, after applying the BH correction, sex was no longer statistically significant. All other predictors, including session number, baseline depression severity, family accommodation, OCD severity at baseline, OCD insight, and age were not significantly associated with homework adherence, *ps* > 0.05. Results for all tested predictors are presented in Table 4.

## 3. Discussion

Unfortunately, front-line evidenced-based treatments for pediatric OCD are not sufficiently effective for all youth (Kemp et al., 2021; McGuire et al., 2015). Our findings highlight that homework adherence is an important therapeutic target of exposure-based CBT that can lead to improved clinical outcomes. Results revealed a significant relationship between homework adherence and reduced OCD severity, increased incidence of a treatment response, and clinical remission at the post-treatment assessment. Notably, the presence of co-occurring ADHD was identified as the strongest factor corresponding with lower homework adherence in exposure-based CBT. Taken together, these findings have several clinical implications that provide a pathway to optimize outcomes for youth with OCD.

First, these findings further reinforce the growing literature demonstrating the importance of homework adherence in pediatric OCD treatment outcomes (Olatunji et al., 2015; Park et al., 2014; Selles et al., 2018; Tuerk et al., 2024; Walther et al., 2022). The current study highlights that both average homework adherence and within person session-by-session increases predict reductions in symptom severity across treatment. Additionally, late homework adherence (as opposed to early homework adherence) is most salient in predicting positive treatment outcomes. Homework assignments early in treatment typically relate to psychoeducation, hierarchy development, and cognitive strategies. Meanwhile homework assignments later in treatment predominately consist of exposures. Consequently, therapists should emphasize the between-session completion of exposures in CBT as it has implications to positively influence treatment outcomes.

Given the established link between homework adherence and treatment outcomes in pediatric OCD, further research is critical to identify strategies to optimize homework adherence. These strategies may include: (1) automated reminders to practice exposures during the week, (2) utilization of behavioral reward systems, and (3) greater parental involvement—all of which have shown promise in increasing engagement in related evidence-based treatments (Jones et al., 2021; Klein et al., 2024). Beyond this, innovative technologies have shown promise to improve homework adherence (Klein et al., 2024; Silk et al., 2020; Tang & Kreindler, 2017; Tuerk et al., 2024). Integrating therapeutic strategies alongside easy to learn and use applications can help improve homework adherence and optimize clinical outcomes from exposure-based CBT.

When developing strategies to optimize homework adherence in clinical practice, it is important to consider the effects of ADHD. Youth with ADHD may require more frequent reminders and greater supervision to complete homework (Farrell et al., 2020). Consequently, prior or concurrent treatment of ADHD through pharmacotherapy and/or behavioral treatment may be essential to improve adherence and outcomes in youth (Geller et al., 2002; Sukhodolsky et al., 2013). On balance, there are mixed findings as to whether ADHD entirely attenuates treatment response (McGuire et al., 2015; Storch et al., 2008). Further research is needed to understand the potential mechanisms linking ADHD to lower homework adherence and ultimately therapeutic improvement. Overall, a thorough assessment and strategizing early in exposure-based CBT may help to decrease barriers and ultimately improve adherence and clinical outcomes.

Despite the strengths of this study that include the largest sample size to date, rigorous clinical trial methodology, utilization of clinician-rated OCD assessments, and fidelity to evidence-based treatment (Storch et al., 2016), some limitations do exist. First, assessment of homework adherence was limited by the measurement and reliance on subjective clinician decisions. Future research would benefit from replication with studies implementing a more detailed measure of adherence (e.g., Patient Adherence Scale for ERP; Simpson et al., 2010), or use of objective mHealth measures of adherence (e.g., OC-Go; Tuerk et al., 2024). This would provide a multimodal approach to better characterize and understand findings related to homework adherence. Second, the reason for homework non-adherence was not documented. In practice, there may be multiple reasons for homework non-adherence. While the current paper identified clinical barriers to homework adherence, additional investigations should consider the environmental barriers to adherence, including social determinants of health. Finally, the sample had limited racial diversity. This is often the case with many OCD studies (Williams et al., 2010, 2012), so future research with a more diverse sample may provider greater insight into specific external barriers to homework completion and inform strategies to enhance adherence.

Overall, our findings highlight the importance of homework adherence and its direct relationship to positive OCD treatment outcomes. There remains a critical need for future research to optimize treatment gains by assessing new adherence strategies in larger clinical trials. Indeed, preliminary results are promising given evidence to suggest that homework adherence can be modified to improve treatment outcomes (Klein et al., 2024; Tuerk et al., 2024). The further development of interventions to target homework adherence can help to achieve greater treatment benefits among youth most in need.

## Figures and Tables

**Table 1:** Descriptive statistics and zero-order correlations among study variables.

Variable	Mean	SD	1	2	3	4	5	6	7	8	9	10
1. Average homework adherence	4.10	0.96	-									
2. Early homework adherence	4.15	1.08	**0.89**									
3. Late homework adherence	4.06	1.03	**0.92**	**0.63**								
4. CY-BOCS baseline	25.28	5.98	−0.14	−0.14	−0.09							
5. CY-BOCS post-treatment	13.87	6.98	−**0.27**	−0.11	−**0.36**	**0.36**						
6. CDRS baseline	26.00	8.70	0.003	0.02	0.01	**0.34**	**0.30**					
7. FAS baseline	15.03	8.78	−0.06	0.02	−0.10	**0.27**	**0.27**	0.12				
8. OCD insight	1.15	1.03	−**0.24**	−0.16	−**0.23**	**0.23**	0.08	−0.1	0.09			
9. Age	12.42	2.88	−0.04	−0.06	−0.02	−0.01	0.11	**0.22**	−**0.19**	−0.16		
10. ADHD Diagnosis	-	-	−0.15	−0.11	−0.16	−0.01	0.13	0.04	**0.21**	0.13	−0.08	
11. Sex	-	-	−**0.17**	−**0.18**	−0.11	−0.03	0.04	−0.04	0.02	0.11	0.02	0.1

Note. Bold values indicate p < .05.

**Table 2:** Mixed-effects linear model predicting CY-BOCS scores from homework adherence.

Predictor	β	SE	95% CI	*p*
Fixed Effects				
Intercept	0.00	2.50	[0.00, 0.00]	<0.001
Person-Centered Homework	0.05	0.03	[−0.03, 0.14]	0.23
Homework Mean	−0.03	0.06	[−0.19, 0.11]	0.68
Session	−0.20	0.12	[−0.44, 0.05]	0.12
Person-Centered Homework x Session	−0.05	0.02	[−0.09, −0.01]	0.02
Homework Mean x Session	−0.07	0.03	[−0.13, −0.01]	0.02

Note. β = standardized estimate; SE = standard error; 95% CI = confidence interval.

**Table 3:** Mixed-effects linear model predicting CY-BOCS scores from early and late homework adherence.

Predictor	β	SE	95% CI	*p*
Fixed Effects				
Intercept	0.00	2.41	[0.00, 0.00]	<0.001
Early Homework Adherence	−0.10	0.07	[−0.28, 0.08]	0.29
Late Homework Adherence	0.02	0.07	[−0.16, 0.21]	0.81
Session	−0.27	0.12	[−0.51, −0.03]	0.03
Early Homework Adherence x Session	0.06	0.04	[−0.01, 0.13]	0.07
Late Homework Adherence x Session	−0.13	0.04	[−0.20, −0.06]	<0.001

Note. β = standardized estimate; SE = standard error; 95% CI = confidence interval.

**Table 4:** Mixed-effects linear model predicting homework adherence across treatment sessions from baseline clinical and demographic characteristics.

Predictor	β	SE	95% CI	*p*
Fixed Effects				
Intercept	0.00	0.55	[0.00, 0.00]	<0.001
Session	−0.03	0.01	[−0.08, 0.02]	0.26
Baseline Depressive Symptoms	0.03	0.01	[−0.10, 0.16]	0.65
Baseline Family Accommodation	0.01	0.01	[−0.11, 0.13]	0.89
ADHD Diagnosis	−0.14	0.20	[−0.26, −0.02]	0.02
Baseline OCD Severity	−0.09	0.02	[−0.22, 0.04]	0.20
CY-BOCS Insight	−0.11	0.08	[−0.23, 0.01]	0.08
Age	−0.07	0.03	[−0.18, 0.05]	0.28
Sex	−0.12	0.16	[−0.23, −0.01]	0.04

Note. β = standardized estimate; SE = standard error; 95% CI = confidence interval.

## Data Availability

Data will be made available upon reasonable request to the corresponding author.
